# Van der Waals Integration of Tungsten Diselenide-Based Radio-Frequency Switches Exceeding GHz

**DOI:** 10.34133/research.1359

**Published:** 2026-07-28

**Authors:** Yiguo Liu, Nuo Cheng, Zhehan Wang, Chengdong Zhao, Xuyan Zhang, Dong Li, Zixiang Mao, Zijian Jiang, Bowen Wang, Yan Luo, Chao Zhu, Weixiang Jiang, Yichen Liu, Li Tao

**Affiliations:** ^1^ School of Materials Science and Engineering, Southeast University, Nanjing 211189, China.; ^2^ School of Integrated Circuits, Southeast University, Nanjing 211189, China.; ^3^ School of Information Science and Engineering, Southeast University, Nanjing 211189, China.

## Abstract

There is a lack of high-frequency electronics based on 2-dimensional p-type transitional metal dichalcogenides, which originates from channel defects during device fabrication and non-optimized metal–semiconductor–insulator interface. Direct metal deposition inevitably induces n-type dominant carrier transport, and high-k dielectric layers cause an elevated carrier concentration enhancing carrier–carrier scattering. Herein, taking tungsten diselenide (WSe_2_) as an example, we employ a polyvinyl alcohol (PVA)-assisted transfer of electrodes to realize WSe_2_ devices with a high carrier mobility and low hole concentration at the same time. This approach circumvents the interfacial defects and lattice distortions caused by atomic bombardment, cluster dynamics, and localized heating of the contact region during direct metal deposition. Compared to polymethyl methacrylate (PMMA) and other transfer media, the water-soluble PVA layer can be removed without organic solvents, leading to high-quality channel and ideal dielectric interface effectively, which suppress Coulomb impurity scattering and carrier–carrier scattering at low hole concentration (~3.5 × 10^12^ cm^−2^). This enables a carrier mobility exceeding 140 cm^2^ V^−1^ s^−1^ at room temperature, superior to other heavily doped counterparts. Radio-frequency switches fabricated by transferring a top electrode exhibit a cutoff frequency of 57.5 GHz with a low series resistance of 25 Ω. This realizes the path loss and signal integrity required by 6G communications, rendering WSe_2_ a highly promising candidate for millimeter-wave applications.

## Introduction

Traditional radio-frequency (RF) switches based on materials such as gallium arsenide (GaAs) and single crystal Si [1–4] face challenges in further miniaturization of devices [5–7] and exhibit high insertion loss in the on state. Two-dimensional (2D) semiconductors with both mechanical flexibility [8–10] and tunable carrier mobility [11] are promising for next-generation RF and microwave electronics. Tungsten diselenide (WSe_2_), a p-type transitional metal dichalcogenide (TMDC), offers an attractive channel material for high-frequency applications due to its relatively high intrinsic carrier mobility (predicted 693 cm^2^ V^−1^ s^−1^) [12] and moderate bandgap [13], which aligns well with high-work-function metals for high hole injection efficiency. Such band alignment facilitates hole injection and mitigates Schottky barrier formation, thereby reducing contact resistance while preserving efficient in-plane carrier transport [14–18].

Despite the intrinsic advantages of 2D TMDCs, the performance of 2D semiconductor–based RF devices is predominantly limited by the quality of metal–semiconductor contacts. Conventional contact strategies such as electron-beam evaporating and sputtering of metal electrodes [19,20] often induce interface disorder, metal-induced gap states, and Fermi-level pinning, which not only degrade the direct–current (DC) characteristics but also severely constrain the high-frequency switching performance [21,22]. For example, gate-tunable rectification and breakdown characteristics of the device can be achieved through material optimization and contact engineering [23,24]; however, p-type switches still face persistent challenges in simultaneously achieving tunable Schottky barriers, low contact resistance, and high-frequency performance. Recent studies have shown that van der Waals (vdW) metal–semiconductor junctions formed by laminating prefabricated metals onto 2D semiconductors can suppress Fermi-level pinning and enable work-function-tunable carrier injection [21]. Optimized high-work-function metal contacts and hexagonal boron nitride‌ (hBN)-encapsulated transferred via contacts have further enabled high-performance p-type TMDC transistors with reduced contact resistance and improved interface quality [25], reducing interfacial strain, roughness, and contamination of PMMA-assisted wet etching methods [26,27]. Moreover, liquid-metal-oxide-assisted transfer and tier-by-tier vdW lamination have further advanced the concept of prefabricated-stack integration, enabling atomically smooth dielectric/contact interfaces and low-temperature monolithic 3D integration without degrading underlying 2D devices [28]. Existing strategies remain constrained by process complexity and transfer-induced interface degradation, making clean, simple, and damage-free electrode transfer still challenging for future 2D device integration [29–31].

In this work, we extend the residue-reduced clean-transfer concept [32] to prefabricated Pd-electrode integration for p-type WSe_2_ vertical RF switches. Through PVA-assisted transfer, a preserved vdW Pd/WSe_2_ contact interface is established without direct metal deposition damage, thereby extending transferred electrode contact engineering to WSe_2_-based devices. This damage-free contact formation preserves the intrinsic transport properties of WSe_2_, as evidenced by field-effect transistors (FETs) exhibiting a carrier mobility as high as 140.6 cm^2^ V^−1^ s^−1^ at a carrier density of ~3.5 × 10^12^ cm^−2^. We further demonstrate a ground–signal–ground (GSG) RF switch based on the optimized WSe_2_ device architecture. The device exhibits a low series resistance of 25 Ω, together with a return loss of −1.65 dB and an isolation of −15.7 dB within an operating frequency band up to 57.5 GHz. This work provides a unified framework for preserving channel integrity while enabling efficient RF switching, offering a promising route toward high-performance 2D communication devices.

## Results and Discussion

### Device architecture and channel-protected fabrication strategy

**Fig. 1.**
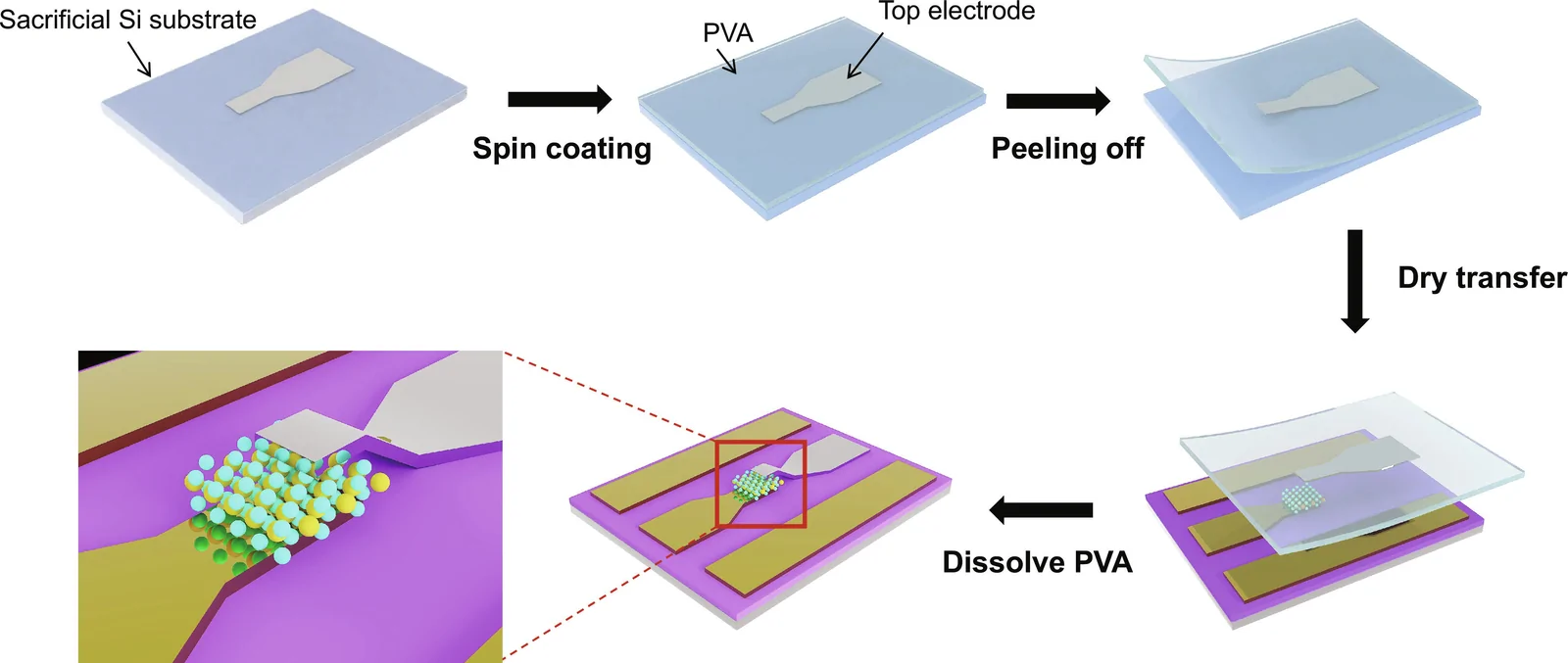
Schematic illustration of the transfer process. The pre-fabricated metal electrodes are first prepared on a sacrificial substrate using lithography and metal deposition processes. PVA layer is spin-coated onto the electrode-bearing substrate and thermally cured to form a mechanically stable support film.

Figure 1 illustrates the schematic of device architecture and the PVA-assisted transfer process for mechanically exfoliated WSe_2_ (Fig. S1). The proposed structure features a vertically stacked configuration in which a prepatterned metal electrode (Fig. S2) is laminated onto the WSe_2_ channel via a vdW assembly approach, rather than being directly deposited onto the semiconductor surface. The PVA support layer was dissolved in deionized water, resulting in the intimate vdW contact between the prepatterned metal electrode and the underlying WSe_2_ surface. This water-assisted removal process avoids aggressive solvents and energetic metal deposition, thereby preserving the lattice integrity and interfacial quality of the atomically thin WSe_2_ channel. Compared with electrode fabrication by electron-beam evaporation, this electrode transfer method enables superior formation of high-quality vdW contacts at the metal/WSe_2_ interface and preserves the intrinsic electrical properties of 2D WSe_2_ nanosheets, as detailed in the following discussion.

**Fig. 2.**
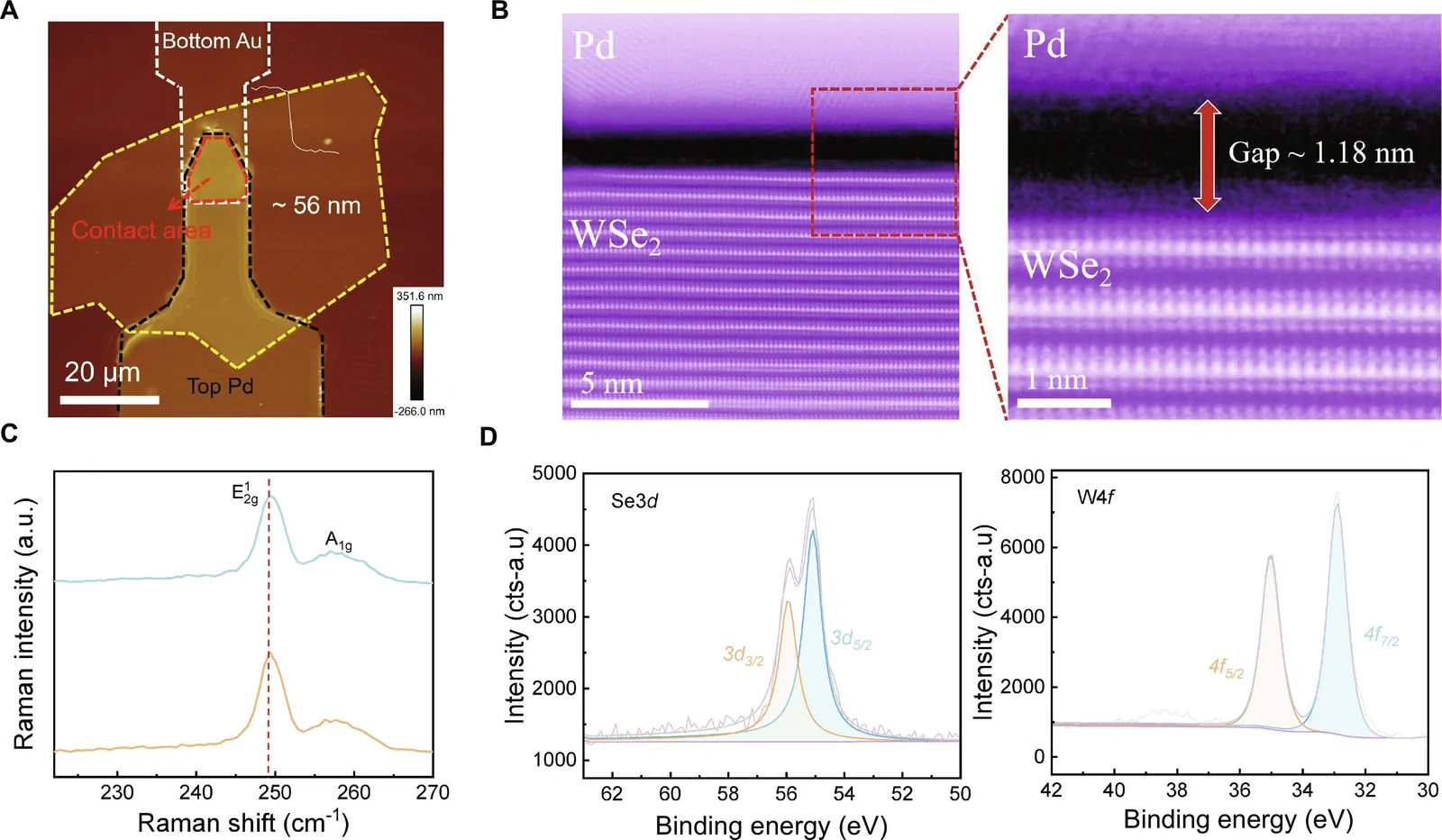
Schematic diagram and characterization of vdW contact structure. (A) AFM image of the WSe_2_ vertical Schottky diode. (B) STEM image of vdW contact between metal Pd and WSe_2_. (C) Raman spectra of WSe_2_ before (blue) and after (orange) electrode transfer. (D) XPS spectroscopy characterization of WSe_2_ for device fabrication.

Figure 2A illustrates the atomic force microscopy (AFM) image of a WSe_2_ vertical Schottky diode. A relatively thick WSe_2_ flake was intentionally selected for the vertical RF Schottky diode, because this device geometry requires not only a clean vdW contact interface but also a sufficiently robust out-of-plane transport pathway. Compared with monolayer or few-layer WSe_2_, thicker flakes provide a larger vertical conduction volume, better mechanical stability during device fabrication, and reduced device-to-device variability. More importantly, the increased transport volume can help suppress parasitic series resistance and stabilize the current pathway, which is beneficial for obtaining reliable current modulation and measurable high-frequency response in RF diode operation [24,33–35]. During device fabrication, the bottom ohmic contact electrode consisting of Au/Ti (20/5 nm) was first patterned by electron-beam evaporation. A 56-nm-thick WSe_2_ flake was then mechanically exfoliated and transferred onto the bottom electrode/SiO_2_ substrate using a 2D material transfer platform, thereby minimizing interfacial defects and forming a high-quality WSe_2_/SiO_2_ dielectric interface. Then, a 50-nm-thick Pd electrode that overlaps the bottom electrode of a 102-μm^2^ area is patterned. The device pads are designed in a GSG configuration to achieve a 50 Ω characteristic impedance for RF measurements. To investigate the atomic structure of the obtained interface, scanning transmission electron microscopy (STEM) was performed to investigate the atomic structure at the WSe_2_–Pd cross-section, as depicted in Fig. 2B. The layered WSe_2_ lattice and the bottom boundary of the transferred Pd electrode can be more clearly distinguished. The vdW gap was determined by measuring the vertical distance between the topmost Se atomic plane of WSe_2_ and the nearest Pd atomic contrast at the interface. We performed measurements at multiple positions along the interface and obtained an average interfacial spacing of approximately ~1.18 nm. To assess whether the PVA-assisted transfer process preserves the structural integrity of the WSe_2_ channel, Raman spectroscopy was performed before and after electrode transfer. Figure 2C shows the characteristic Raman modes of mechanically exfoliated WSe_2_, including the in-plane $E_{2g}^{1}$ and out-of-plane $A_{1g}$ vibrational modes. Notably, the peak positions and full width at half maximum remain nearly unchanged after the vdW electrode transfer, indicating that no important strain, lattice distortion, or defect-induced phonon scattering is introduced during the lamination process. Meanwhile, there are no overlaps of miscellaneous peaks related to any organic functional groups, indicating the absence of PVA residues or other organic contaminants in the tested region. Unlike PMMA-assisted transfer [36], the water-soluble PVA layer can be removed without organic solvents, significantly suppressing polymer residues. X-ray photoelectron spectroscopy (XPS) measurements were performed on the WSe_2_ layer for device fabrication in Fig. 2D. The corresponding Se 3d and W 4f spectra revealed that the characteristic peak positions of 2H-WSe_2_ (W 4f₅/₂ and W 4f₇/₂ at 35.08 and 32.88 eV, respectively; Se 3d₃/₂ and Se 3d₅/₂ at 55.98 and 55.08 eV, respectively) showed no shift within the experimental resolution. This result confirms the formation of a weakly coupled vdW contact between the electrode and WSe_2_ film, with no chemical reaction occurring.

### Electrical transport behavior of channel-protected WSe_2_ FETs

**Fig. 3.**
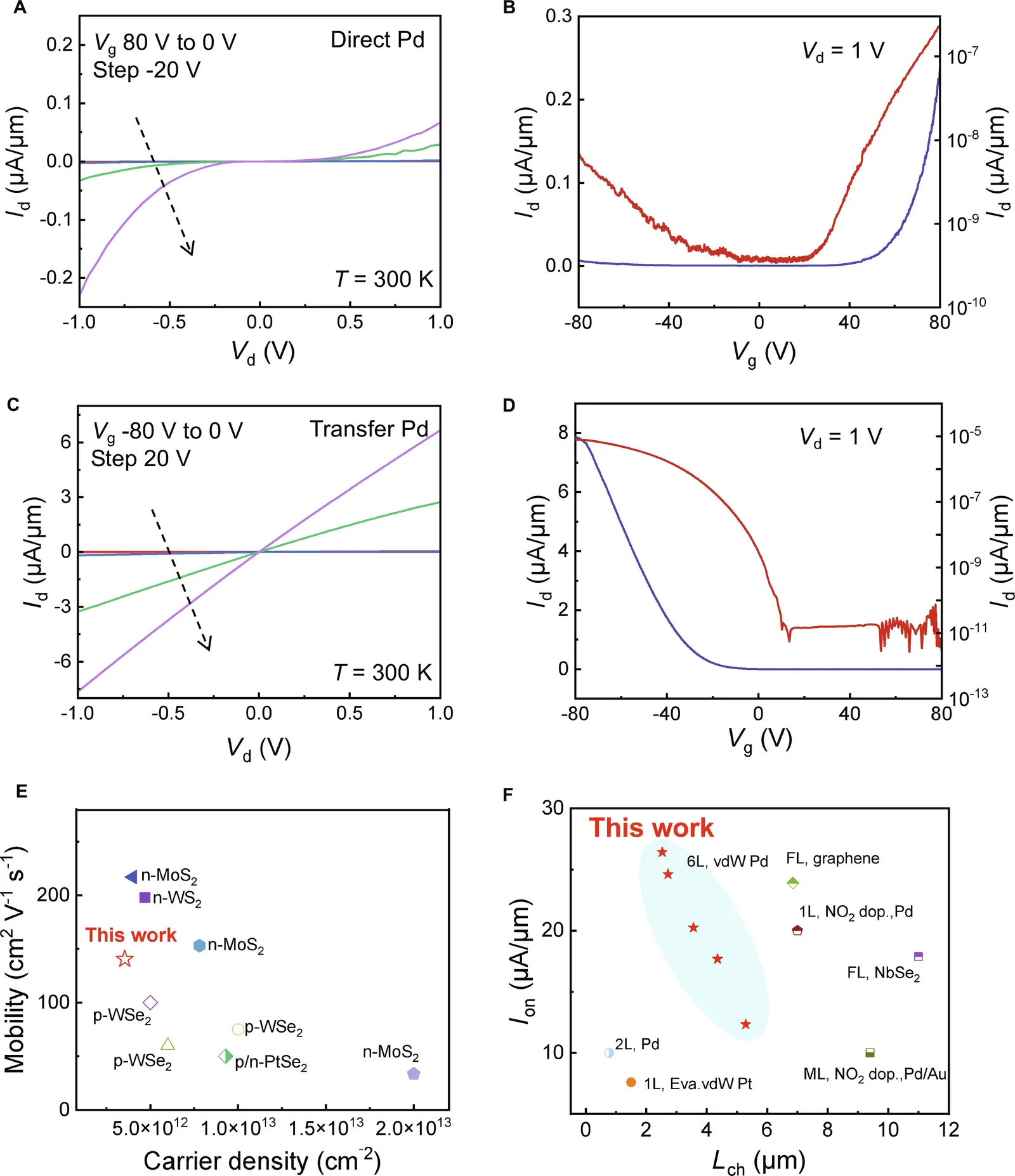
(A and B) Output and transfer characteristics of WSe_2_ FETs with direct deposited Pd contacts. (C and D) Output and transfer characteristics of WSe_2_ FETs with vdW Pd contacts. (E) Mobility versus carrier density of reported n-type (filled) and p-type (empty) TMDC FETs [39–46] . (F) Saturation on-state current density (*I*_on_) as a function of *L*_ch_ of WSe_2_ FETs with various contact technologies reported in the literature [25,49–53].

High-work-function metals such as Pd are preferred for contacting WSe_2_ because their Fermi level aligns more closely with the valence band maximum, enabling efficient hole injection and reduced Schottky barrier height (SBH) [9]. However, during electron-beam evaporation of Pd, high-energy metal atoms strongly bombard the WSe_2_ surface, introducing a large number of selenium vacancies, lattice defects, and surface dangling bonds. These defects generate a high density of interface states within the bandgap of WSe_2_, leading to pronounced Fermi-level pinning. Wang and colleagues [37] achieved clean vdW interfaces between high-work-function metals such as Pt/Pd and 2D TMDs by optimizing electron-beam evaporation conditions, but this approach requires expensive equipment and involves relatively high fabrication costs. The contact-interface improvement of this work was verified in transistor devices with thin WSe_2_ layers (approximately 4 nm depicted in Fig. S3), which is therefore more sensitive to metal-induced interface damage than the thick film. For a direct comparison, we fabricated few-layer WSe_2_ FETs using 2 different approaches: the directly evaporated contacts (marked as Direct Pd in Fig. 3A and B) and the transferred electrode devices (marked as Transfer Pd in Fig. 3C and D). As shown in Fig. 3A, WSe_2_ FET with directly deposited Pd electrodes exhibit pronounced Schottky rectification behavior and nonlinearity in the output curves. These defect states cause the Fermi level (*E*_F_) to be strongly pinned near the conduction band minimum (*C*_BM_) of WSe_2_, rather than at its valence band maximum (*V*_BM_). Although the work function of Pd (~5.1 eV) is significantly higher than the electron affinity of WSe_2_ (~4.5 eV), this strong pinning effect suppresses hole injection and renders electrons the dominant carriers. Consequently, the transfer characteristics in Fig. 3B confirm the n-type transport behavior. At a gate voltage *V*_g_ of 80 V, the on-state current is only 0.24 μA/μm, and the field-effect mobility is 12.95 cm^2^ V^−1^ s^−1^.

In contrast, the clean interface significantly weakens the Fermi level pinning effect, enabling high-work-function Pd to achieve good band alignment with the valence band maximum of p-type WSe_2_, thereby greatly reducing the hole injection barrier height. As shown in Fig. 3C, the device exhibits linear and symmetric *I*_d_–*V*_d_ output characteristics under gate voltages *V*_g_. The transferred Pd electrodes are fabricated using a pre-made electrode stacking method, which avoids damage to the WSe_2_ surface caused by high-energy particles, substantially reducing carrier scattering centers and preserving the crystal integrity of WSe_2_ as well as its intrinsic p-type carrier behavior, as illustrated in Fig. 3D. Compared with direct deposition, the device fabricated with PVA-transferred electrodes achieves an on-state current of 7.58 μA/μm with a hole mobility of 140.55 cm^2^ V^−1^ s^−1^ at room temperature, which is significantly higher than the values reported for PMMA-transferred devices (1.2 cm^2^ V^−1^ s^−1^) and polycarbonate (PC)-transferred devices (43 cm^2^ V^−1^ s^−1^) [36]. The SBH [38] of WSe_2_ FET with transferred Pd electrodes is 162 meV (Fig. S4). This improved carrier mobility directly lowers the device's resistance, which in turn significantly optimizes both the insertion loss and isolation performance of the RF switch at high frequencies.

The sheet carrier concentration of state-of-the-art TMDC transistors typically ranges from 10^12^ to 10^13^ cm^−2^ at optimized doping conditions (Fig. 3E) [39–46]. The corresponding hole mobility is significantly influenced by both the carrier density and the dielectric environment. Notably, the observed mobility degradation at elevated carrier concentrations (greater than 5 × 10^12^ cm^−2^) indicates the dominance of phonon scattering mechanisms, consistent with theoretical predictions of anisotropic carrier transport in WSe_2_. The WSe_2_ FETs examined in this work demonstrate a relatively high mobility at low carrier concentrations (~3.5 × 10^12^ cm^−2^), suggesting effective suppression of carrier scattering. 3Figure 3F benchmarks the saturation on-state current density of WSe_2_ p-FETs versus channel length, showing that vdW Pd contacts allows short-channel WSe_2_ devices to deliver a high *I*_on_ of 26.42 μA/μm at *L*_ch_ ~ 2.5 μm, outperforming most reported WSe_2_ contact technologies at comparable channel lengths. Figure S5 depicts the transfer characteristics of WSe_2_ FETs with different channel lengths in detail.

### RF performance of WSe_2_ devices

**Fig. 4.**
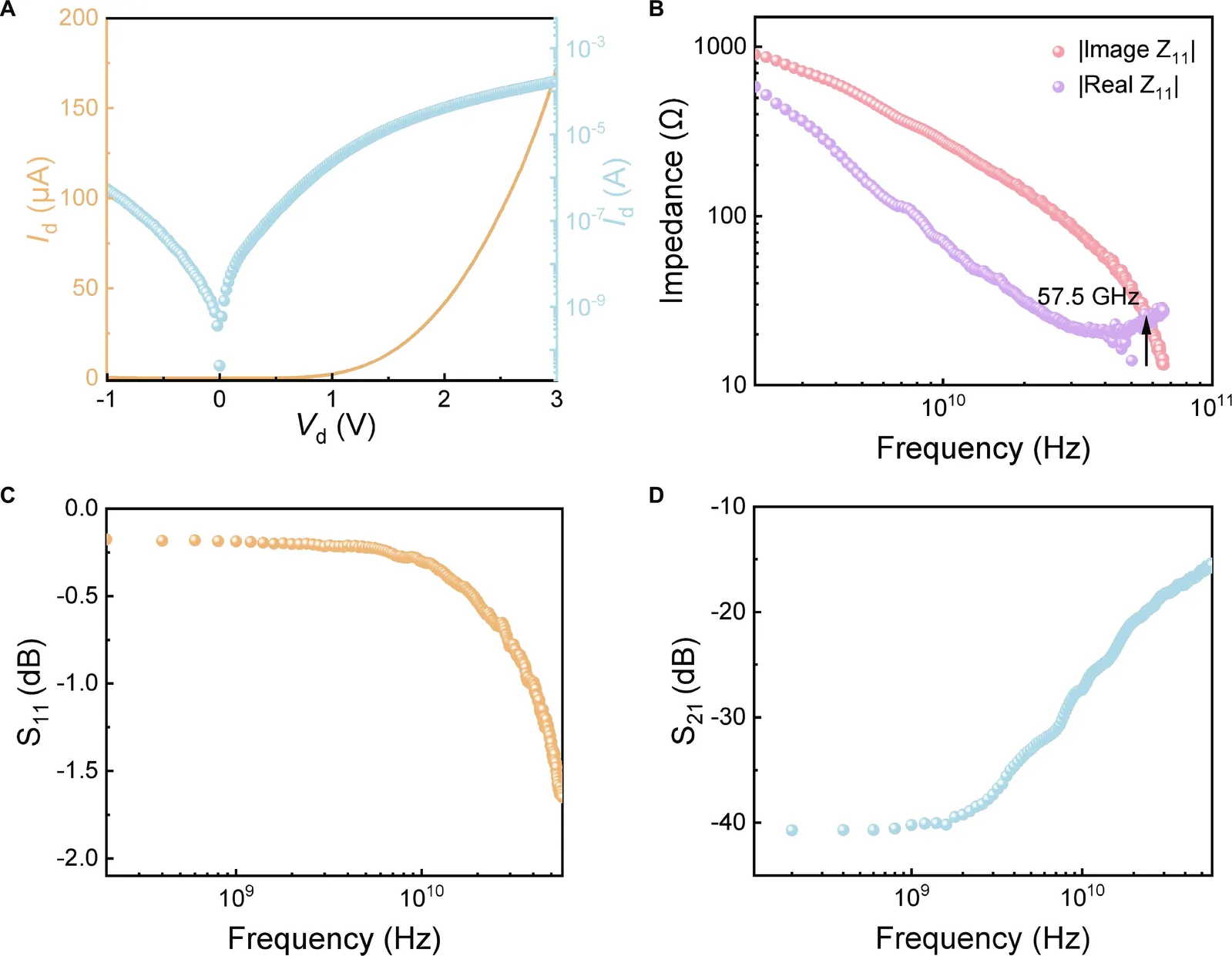
WSe_2_-based radio-frequency (RF) switches. (A) Rectifying characteristics of the RF switch. Inset: Schematic cross-section of the vertical Schottky diode. (B) Cutoff frequency of the RF switch. (C) S_11_ for the WSe_2_ RF switch at 0.2 to 57.5 GHz. (D) S_21_ for the WSe_2_ RF switch at 0.2 to 57.5 GHz.

In the vertical RF switch geometry, an optimized electrode overlap area and WSe_2_ thickness are essential for balancing the series resistance and junction capacitance. A thicker WSe_2_ layer can improve the breakdown robustness of the device and thereby enhance its overall operational stability and performance. To investigate the RF performance enhancement from geometry parameters, we have fabricated devices with an electrode overlap area of 102 μm^2^ and material thickness of 56 nm. We first measured the current–voltage (*I*–*V*) characteristics of WSe_2_ switch, which are shown in Fig. 4A, exhibiting typical rectifying diode behavior. At |*V*_d_| = 3 V, the device shows pronounced current asymmetry, with a forward current of *I*_d_ = 183 μA and significantly lower reverse current. To further evaluate the diode transport characteristics, the ideality factor was extracted from the forward-bias region using the Shockley equation, as shown in Fig. S6. Figure 4B presents the frequency-dependent series resistance (*R*_s_) and capacitive reactance (|*X*_c_| = 1/ω*C*_tot_) under zero DC bias (*V*_DC_ = 0 V). Here, the real part of the measured input impedance, |Re(Z_11_)|, directly reflects the series resistance *R*_s_, while the magnitude of the imaginary part, |Im(Z_11_)|, represents the total capacitive reactance |*X*_c_|, with *C*_tot_ = *C*_j_ + *C*_ox_. The intersection point of |Re(Z_11_)| and |Im(Z_11_)| defines the cutoff frequency of the RF switch. It is expected from the plots that the diode with 56-nm-thick WSe_2_ has a cutoff frequency of about 57.5 GHz, and such a high cutoff frequency stems from extremely low *R*_s_ value (~25 Ω), mostly of contact resistance. The small *R*_s_ provides evidence that the PVA-assisted transferred electrode forms a low-damage and electrically favorable contact interface, which facilitates carrier injection and reduces RF conduction loss. To elucidate the impact of WSe_2_ flake thickness on device performance, we characterized a vertical RF switch fabricated with 17-nm and 20-nm-thick WSe_2_ flakes. As shown in Fig. S7 and summarized in Table S1, the thicker WSe_2_ device exhibits superior performance, including enhanced breakdown robustness and more stable current modulation, compared with thinner flakes. These results confirm that increasing the vertical transport volume not only mitigates device failure under high bias but also improves overall RF switching characteristics, highlighting the importance of flake thickness optimization for high-frequency WSe_2_ devices.

The RF performance of the WSe_2_-based switch was characterized by analyzing the S-parameters, including S_11_ (return loss) and S_21_ (isolation). S-parameter measurements were performed using a VNA (vector network analyzer), which were carefully evaluated and are plotted in Fig. 4C and D. A low return loss of about −1.65 dB and an isolation of −15.7 dB were measured at the cutoff frequency . The on-state insertion loss is −4.8 dB, as presented in Fig. S8, indicating minimal signal attenuation and efficient transmission.

**Fig. 5.**
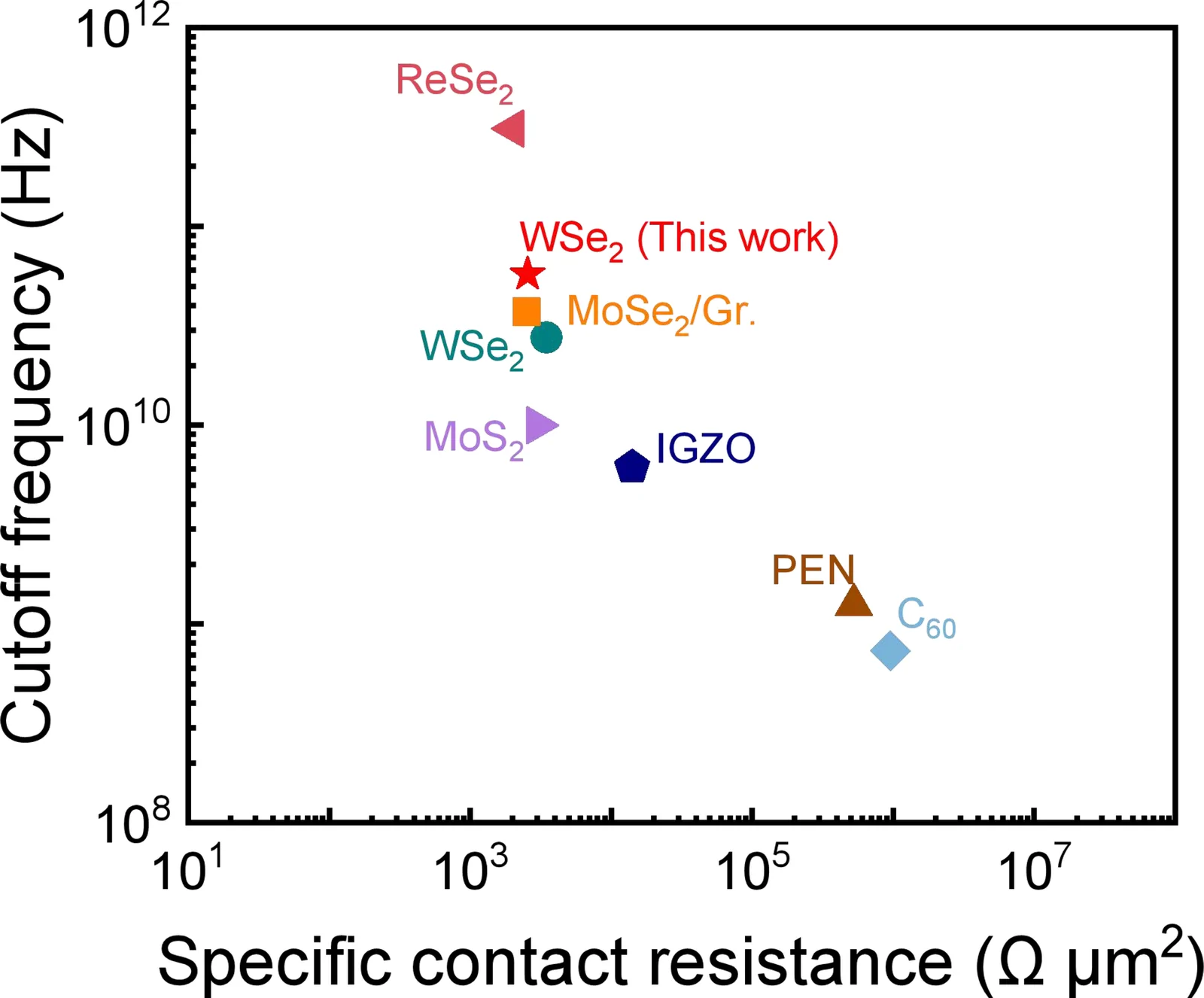
Benchmark comparison of cutoff frequency and specific contact resistance among reported RF switches [19,24,35,47,54–56].

The WSe_2_ RF switch in this work achieves a cutoff frequency of 57.5 GHz and a specific contact resistance of 2,550 Ω·μm^2^ (25 Ω × 102 μm^2^), placing it in the upper-middle performance range among reported RF switches. As summarized in Fig. 5, our device shows a favorable trade-off between high-frequency operation and contact resistance compared with representative switches. In particular, it exhibits a higher cutoff frequency than several reported MoS_2_ and WSe_2_ switches while maintaining a relatively low specific contact resistance. In addition, Table S2 provides a further comparison of other key performance metrics, including the on/off ratio and junction capacitance, for representative graphene- and TMDC-based RF switches reported in the literature [19,24,33,35,47,48].

## Conclusion

This work explores a universal and reliable contact strategy for p-type transition metal dichalcogenides, addressing the critical trade-off between carrier mobility and both return loss and isolation in conventional deposited electrodes. Our results demonstrate that the PVA-assisted vdW transfer of contact electrodes can significantly enhance the hole mobility over 140 cm^2^ V^−1^ s^−1^ in WSe_2_ device. This originates from less interface defects and coupling at the contact region, which effectively suppresses charge scattering and facilitates hole injection. Moreover, such WSe_2_ devices possess high cutoff frequency of 57.5 GHz for RF switches with a low series resistance of 25 Ω. Our strategy realized high-mobility transport and low loss with high isolation in RF characteristics, which holds great promise for 2D semiconductor-based wearable RF electronics for next-generation 6G communications, internet of things, human–machine interface, and other related applications.

## Methods

### PVA-assisted etch-free transfer method

The optimized 8 wt % polyvinyl alcohol (PVA) aqueous solution is prepared using PVA powder (average *M*_w_ ~ 27.045, Shanghai Aladdin) dissolved in deionized (DI) water under continuous stirring at elevated temperature until fully homogenized. The PVA solution is then spin-coated onto the substrate containing the prefabricated metal electrodes at 1,500 r/min for 10 s; spin coating acceleration is set to 100 r/min. After the PVA layer is coated, the sample is heated at 70 °C for 3 min. The above steps are repeated 5 times. The purpose of this step is to construct a polymer support layer with controllable thickness, uniform mechanical properties, and stress stability.

### Preparation of prefabricated electrodes

After chemical cleaning of the silicon wafer, the source/drain patterns are defined and metal deposition is carried out on the silicon wafer through photolithography evaporation process. Ultraviolet exposure and electron-beam evaporation are commonly used in this work.

Negative photoresist nr9-3000 is spin coated on the surface of the silicon wafer at a speed of 500 r/min for 10 s, with a spin coating acceleration of 5,000 r/min for 30 s. After spin coating, the silicon wafer is placed on a heating table at 110 °C for 3 min to complete the pre-baking operation. The main g-line (436 nm) and i-line (365 nm) are exposed in a high-pressure arc lamp for 27 s. After exposure, the silicon wafer is placed on a heating table at 100 °C for 1 min to complete the post-baking operation. The developer used is Resis Developer RD6, with a development time of 10 s.

After photolithography of the electrode pattern, metal deposition is performed using a high vacuum electron-beam evaporator with a vacuum degree of 5 × 10^−4^ Pa or less. Acetone, isopropanol, and DI water are used to complete the lift-off operation. Figure S9 shows high pattern fidelity of PVA-transferred electrode arrays.

### Preparation of WSe_2_ nanosheets

The WSe_2_ nanosheets involved in this work are mainly prepared by the top-down micromechanical exfoliation method with the assistance of polydimethylsiloxane(PDMS). The specific process is as follows: (a) use tweezers to pick up the 2D WSe_2_ seed crystal on the tape dedicated for micromechanical exfoliation, and paste the tape repeatedly to separate the interlayers of the material; (b) attach the PDMS to the tape and gently push along the diagonal of the PDMS with the back of the tweezers to make the tape fit the PDMS without air bubbles; (c) peel off the PDMS, attach the PDMS with the 2D flake WSe_2_ material to the glass slide, and place it under an optical microscope for observation; (d) after finding the 2D WSe_2_ material that meets the requirements under the microscope, load it on the 2D material transfer platform and slowly lower the PDMS to bring the WSe_2_ into contact with the surface of the substrate. It is heated to 80 °C and kept for 4 min to promote adhesion and interface fitting. The PDMS support layer is slowly lifted to leave the WSe_2_ flakes at the designated position on the substrate, completing the preparation of the 2D material.

### Materials characterization

Raman spectroscopy (alpha300Access) is performed by using 532-nm laser source. Transmission electron microscopy was performed using FEI Talos F200X G2. The cross-sectional STEM image of the Pd/WSe_2_ contact is acquired using a Spectra ultra 300 microscope. XPS is performed using Thermo Scientific K-Alpha system. Noncontact mode AFM (Bruker Dimension Icon) is used for characterization of WSe_2_ flake thicknesses and GSG structure.

### Electrical measurements

Electrical measurements are performed by using a semiconductor parameter analyzer (B1500A). The performance of the GSG RF switch is measured using the CASCADE Summit12000.

## Data Availability

All data needed to evaluate the conclusions in the paper are present in the paper or the Supplementary Materials.
