# Supplementary material for: Van der Waals Integration of Tungsten Diselenide-Based Radio-Frequency Switches Exceeding GHz
Source: Research (Wash D C). 2026 Jul 28;9:1359. doi: 10.34133/research.1359 (PMC13439883; doi:10.34133/research.1359)
Supplement: Supplementary file 1 [file research.1359.f1.pdf]

Van der Waals Integration of Tungsten Diselenide based Radio-Frequency Switches with GHz

YIGUO LIU[1*], NUO CHENG[1*], ZHEHAN WANG[1], CHENGDONG ZHAO[1], XUYAN ZHANG[1], DONG LI[1], ZIXIANG MAO[1], ZIJIAN JIANG[1], BOWEN WANG[1], YAN LUO[2], CHAO ZHU[2], WEIXIANG JIANG[3], YICHEN LIU[1*], LI TAO[1*]

[1]School of Materials Science and Engineering, Southeast University, 211189, China.

[2]School of Integrated Circuits, Southeast University, 211189, China.

[3]School of Information Science and Engineering, Southeast University, 211189, China.

Device and material characterization

Fig. S1 shows an atomic-resolution TEM image of of mechanically exfoliated WSe_2 film. A clear hexagonal lattice arrangement is observed, confirming the high crystallinity of the synthesized WSe_2. The measured lattice spacing of ~ 0.28 nm corresponds well to the (100) plane of WSe_2. The overlaid atomic model further verifies the structural consistency between the experimental image and the expected crystal structure, indicating that the WSe_2 lattice remains intact without observable distortion or defects.

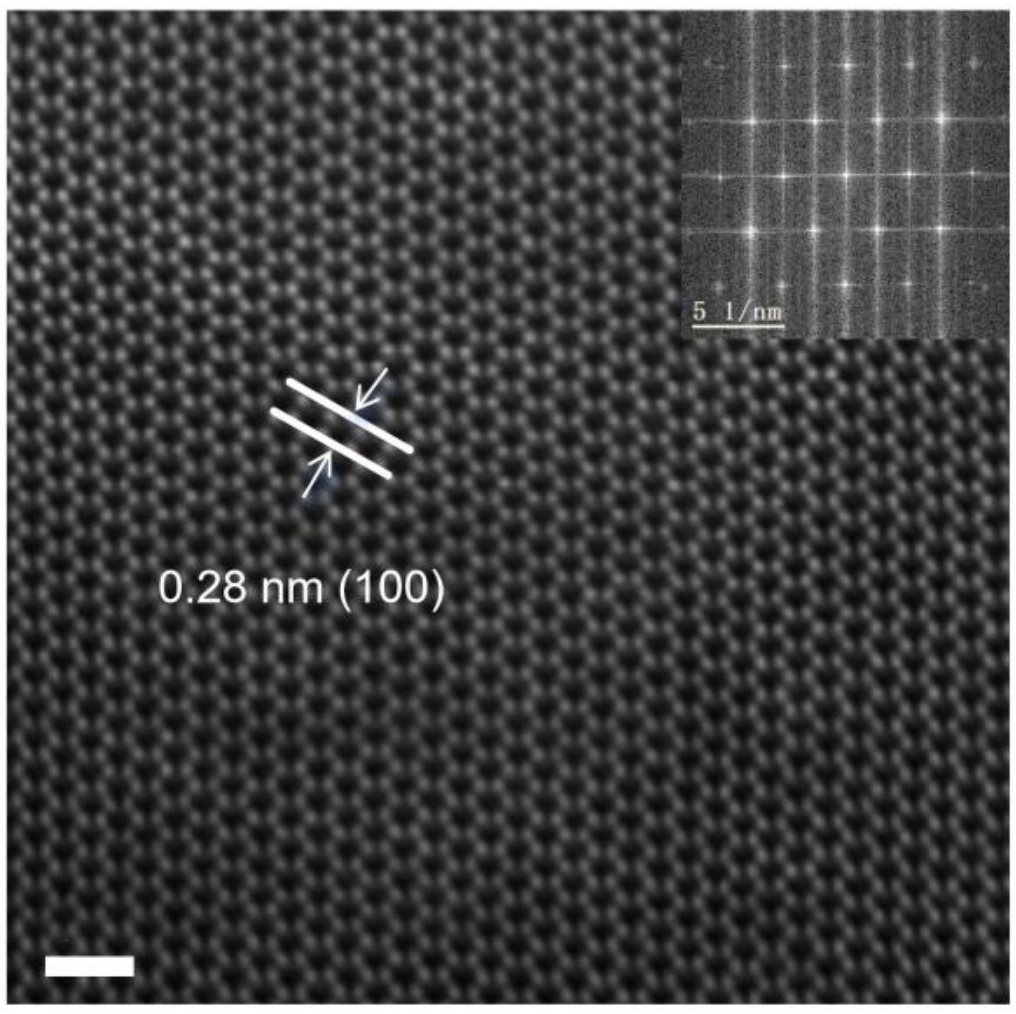

Fig. S1 Planar transmission electron microscope (TEM) image of mechanically exfoliated WSe_2 film (Scale bar, 1 nm).

Fig. S2 illustrates optical microscopy images of the device configuration of the WSe_2 RF switch. In Fig. S2(A), mechanically exfoliated WSe_2 is dry-transferred onto pre-patterned Ti/Au bottom electrodes to form the bottom contact. In Fig. S2(B), a Pd top electrode is transferred onto the WSe_2 flake, resulting in a vertically stacked RF switch architecture.

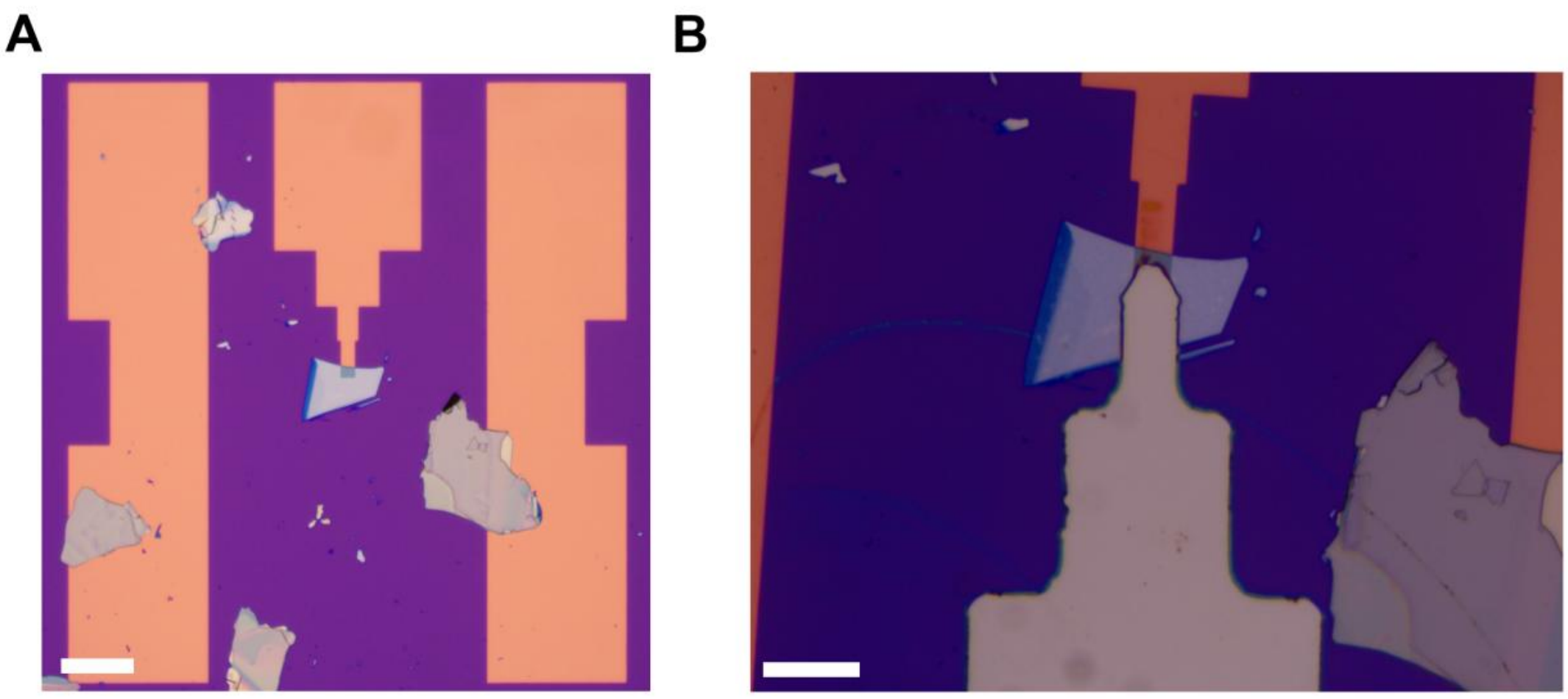

Fig. S2 (A)WSe_2 dry-transferred onto Ti/Au bottom electrodes. (B) Completed device after Pd top-electrode transfer forming a vertical stack (Scale bar, 50 μm).

Fig. S3 depicts atomic force microscopy (AFM) images of the WSe_2 flakes used for different FET configurations. Fig. S3(A) shows a flake for a device with a directly deposited Pd contact, with a measured step height of approximately 4.0 nm. Fig. S3(B) shows a flake for a device with a van der Waals (vdW) transferred Pd contact, exhibiting a similar thickness of ~4.0 nm. These images confirm that the flake thickness is comparable between the two devices, enabling a consistent basis for evaluating contact effects.

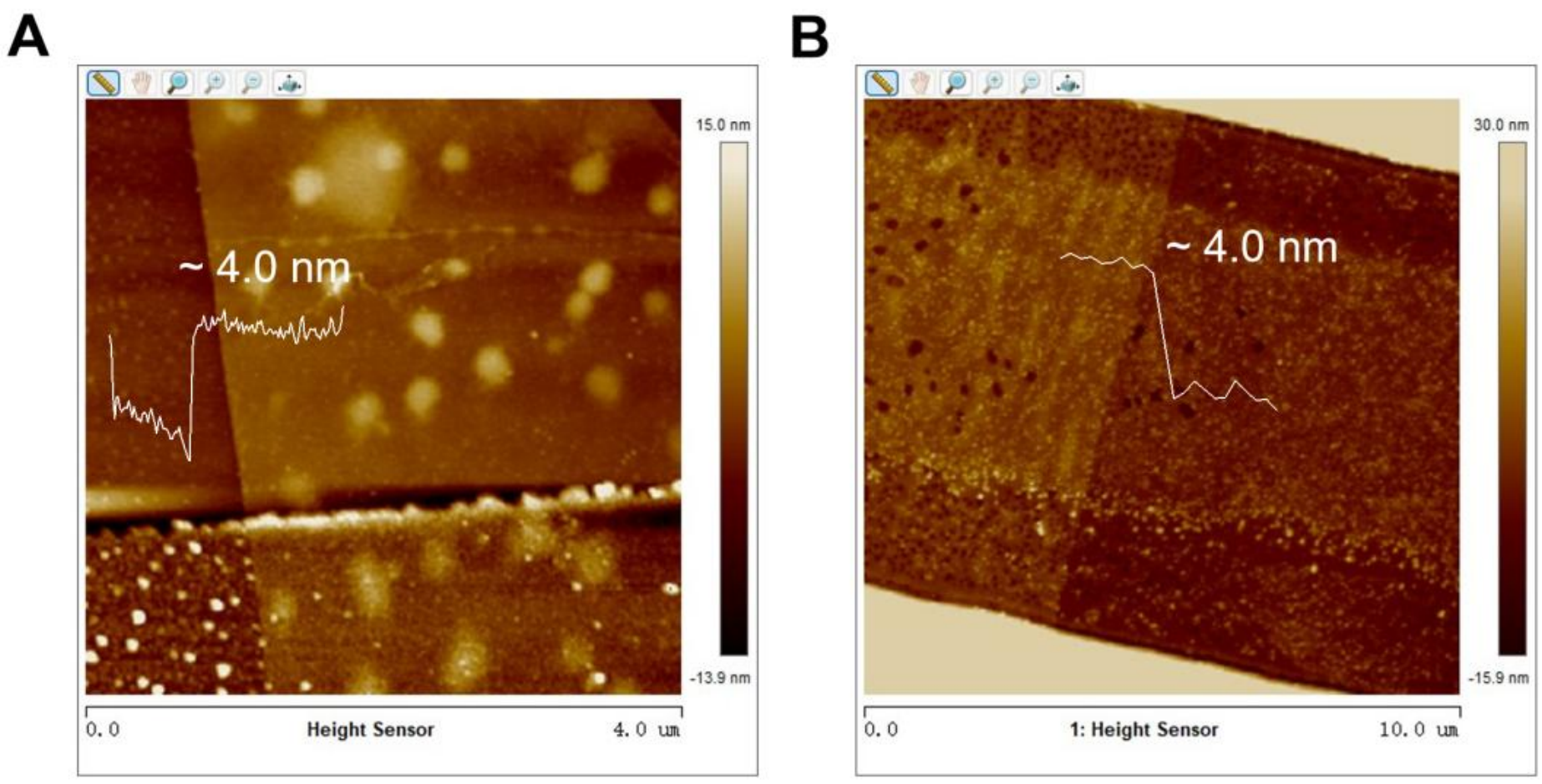

Fig. S3 Thickness of the WSe_2 flakes used for (A) direct deposited Pd contact FET and (B) vdW Pd contact FET

Electrical Characteristics of Field-Effect Transistors

The Schottky barrier height (SBH) of WSe_2 FET with transferred Pd electrodes is 162 meV, which is extracted from equation (2) [38]:

$$I_{DS}=A_{2D}^{*}T^{1.5}\exp\left(-\frac{\Phi_B}{k_BT}\right)\left[1-\exp\left(-\frac{V_{DS}}{k_BT}\right)\right] \tag{2}$$

where $A_{2D}^{*}=q(8\pi k_B^3 m^*)^{0.5}/h^2$ is the Richardson constant for a 2D system (m*, electron effective mass), T is the temperature, k_B is Boltzmann'sconstant, q is the elementary charge, and ΦB is the effective contact barrier height at a given gate-source voltage.

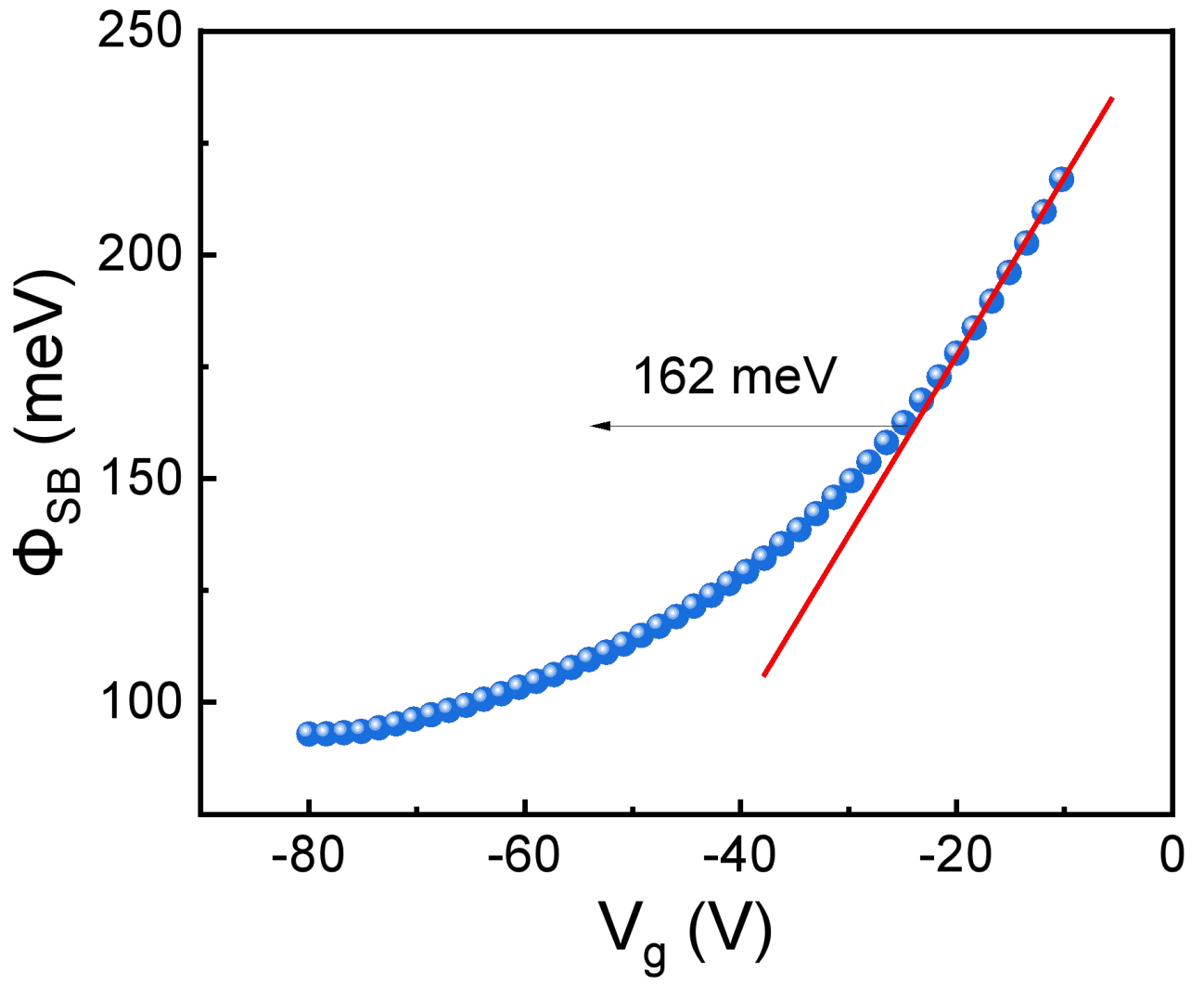

Fig. S4 Schottky barrier extraction of WSe_2 FET with transferred Pd.

Fig. S5 shows the transfer characteristics of WSe_2 FETs with transferred Pd electrodes at different channel lengths. As the channel length decreases from 5.29 µm to 2.52 µm, the normalized drain current increases, with the shortest-channel device exhibiting the highest on-state current density at negative gate bias. All devices show clear p-type transport behavior, indicating efficient hole injection enabled by the transferred Pd contacts.

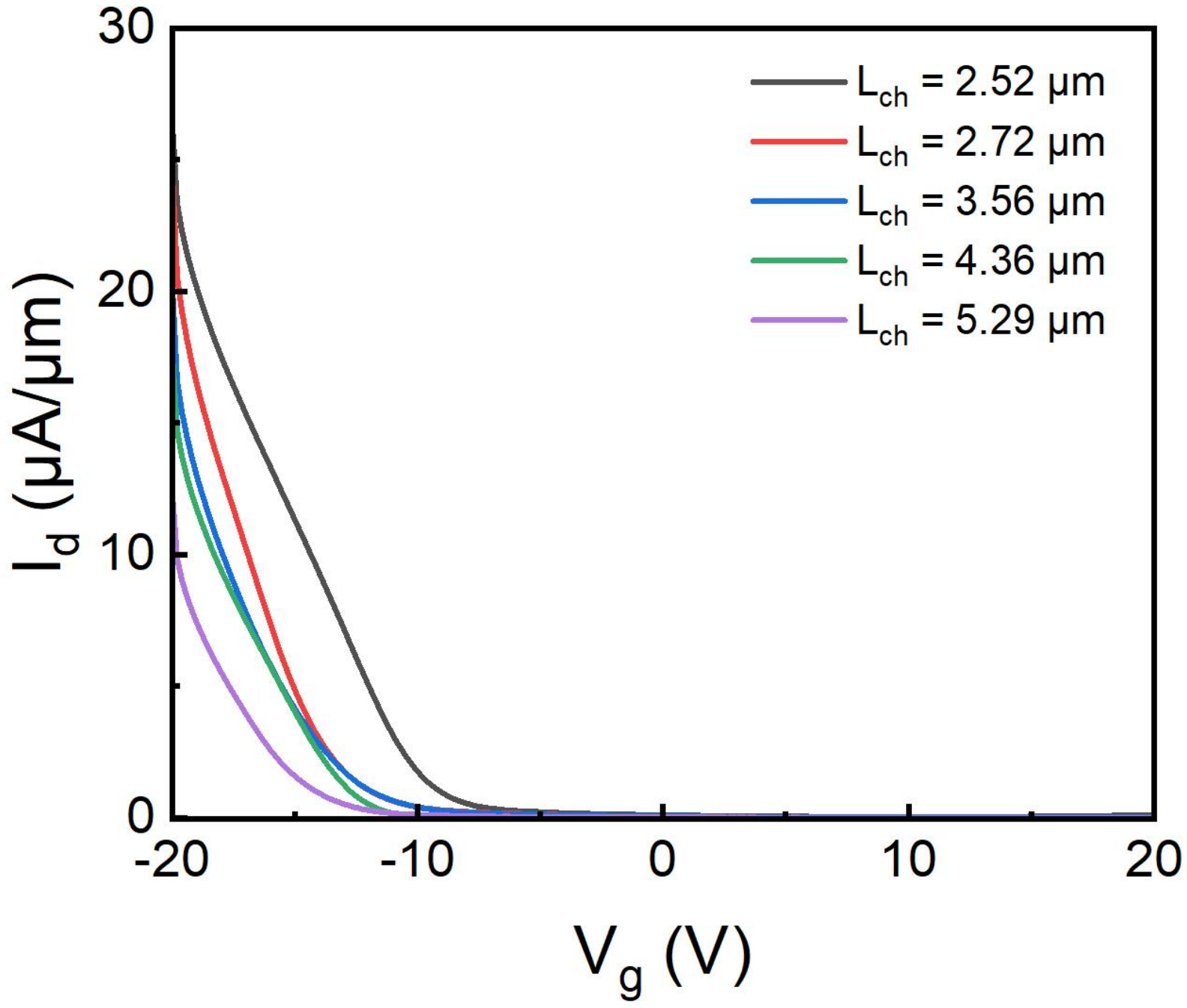

Fig. S5 Transfer characteristics of WSe_2 FETs with different channel lengths.

Performance of WSe_2 Radio-Frequency Switches

The current-voltage (I-V) characteristics of the device can be described by the Shockley equation:

$$I=I_s\left(e^{\frac{qV}{nkT}}-1\right)$$

where I_s is the reverse saturation current, k is the Boltzmann constant, T is the temperature, q is the elementary charge, and n is the ideality factor.

When the applied bias satisfies $V \gg kT/q$, the Shockley equation can be approximated as:

$$I \approx I_s e^{\frac{qV}{nkT}}$$

Taking the natural logarithm gives:

$$\ln I = \ln I_s + \frac{q}{nkT}V$$

Therefore, by performing a linear fitting of the ln(I)-V curve in the forward-bias region, the slope can be expressed as:

$$slope=\frac{\mathrm{d}(\ln I)}{\mathrm{d}V}=\frac{q}{nkT}$$

In this work, the ln(I)-V characteristics in the forward conduction region of the device were linearly fitted. The extracted slope is approximately 8.46, corresponding to an ideality factor n of approximately 4.57.

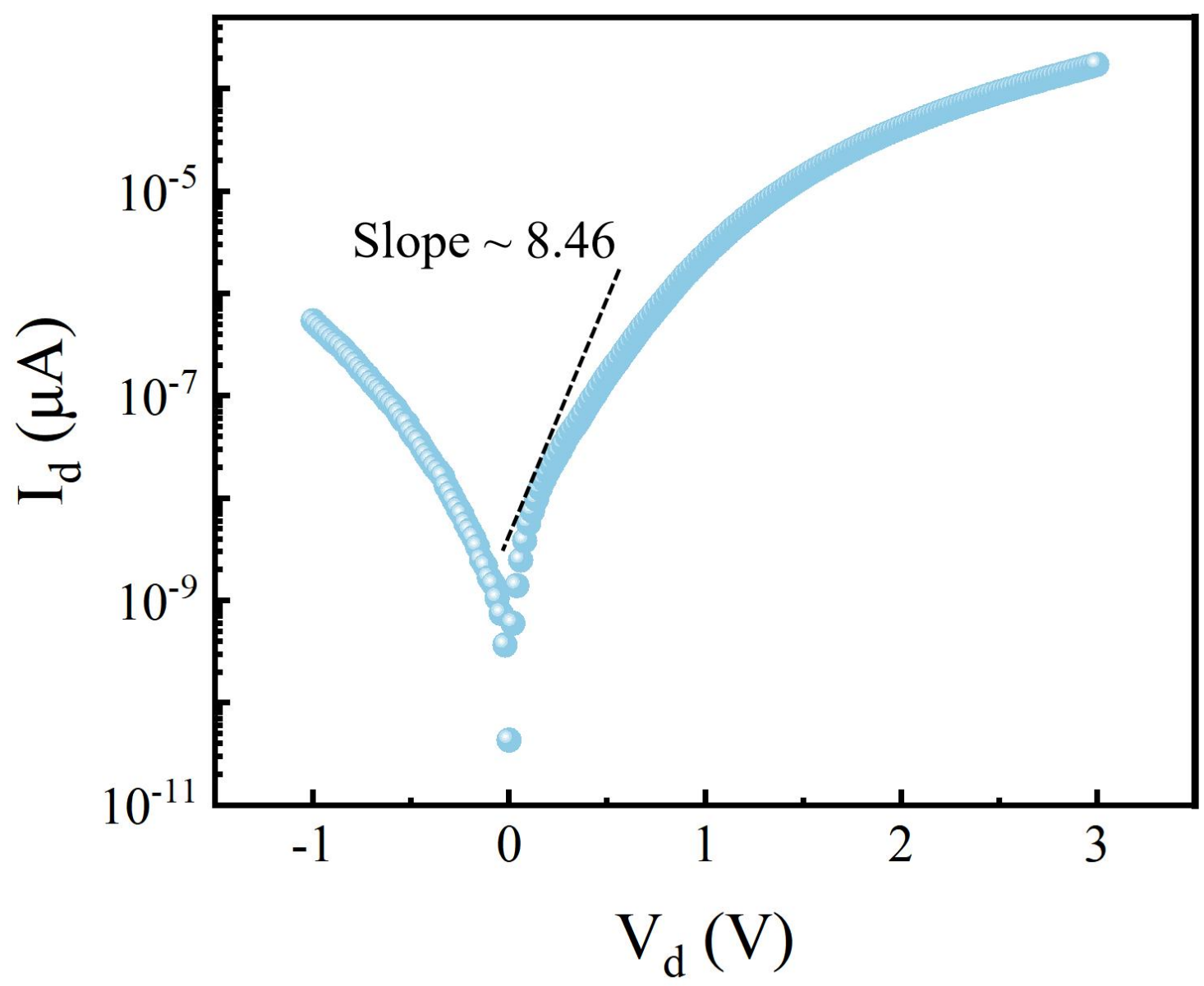

Fig. S6 Quasi-static I-V characteristics of the WSe_2 Schottky diode.

Table S1. Key device parameters of different devices

Parameter	Device 1	Device 2	Device 3
Electrode overlap area	30 μm^2	17.61 μm^2	102 μm^2
Material thickness (t)	17 nm	20 nm	56 nm
Capacitance (Cj)	0.12 pF	0.166 pF	0.11 pF
Normalized junction capacitance	0.004 pF/μm^2	0.009 pF/μm^2	0.001 pF/μm^2
Series resistance	2575 Ω	1068 Ω	25 Ω
Specific contact resistance	77250 Ω·μm^2	18807.48 Ω·μm^2	2550 Ω·μm^2
Electrode symmetry	20/5 nm Au/Ti (bottom electrode) 50 nm Pd (top electrode)	20/5 nm Au/Ti (bottom electrode) 50 nm Pd (top electrode)	20/5 nm Au/Ti (bottom electrode) 50 nm Pd (top electrode)
Cut off frequency	0.5 GHz	0.9 GHz	57.5 GHz

Optical microscope images of Device 1 and Device 2 are shown in Fig. S7(A) and Fig. S7(B), respectively. The corresponding AFM topography images (Fig. S7(C) and Fig. S7(D)) indicate WSe_2 thicknesses of approximately 17 nm and 20 nm. The frequency-dependent real and imaginary components of the input impedance (Z_{11}) for the two devices are presented in Fig. S7(E) and Fig. S7(F), respectively. Device 3 in Table S1 is the RF switch shown in Fig. 4 of the main text.

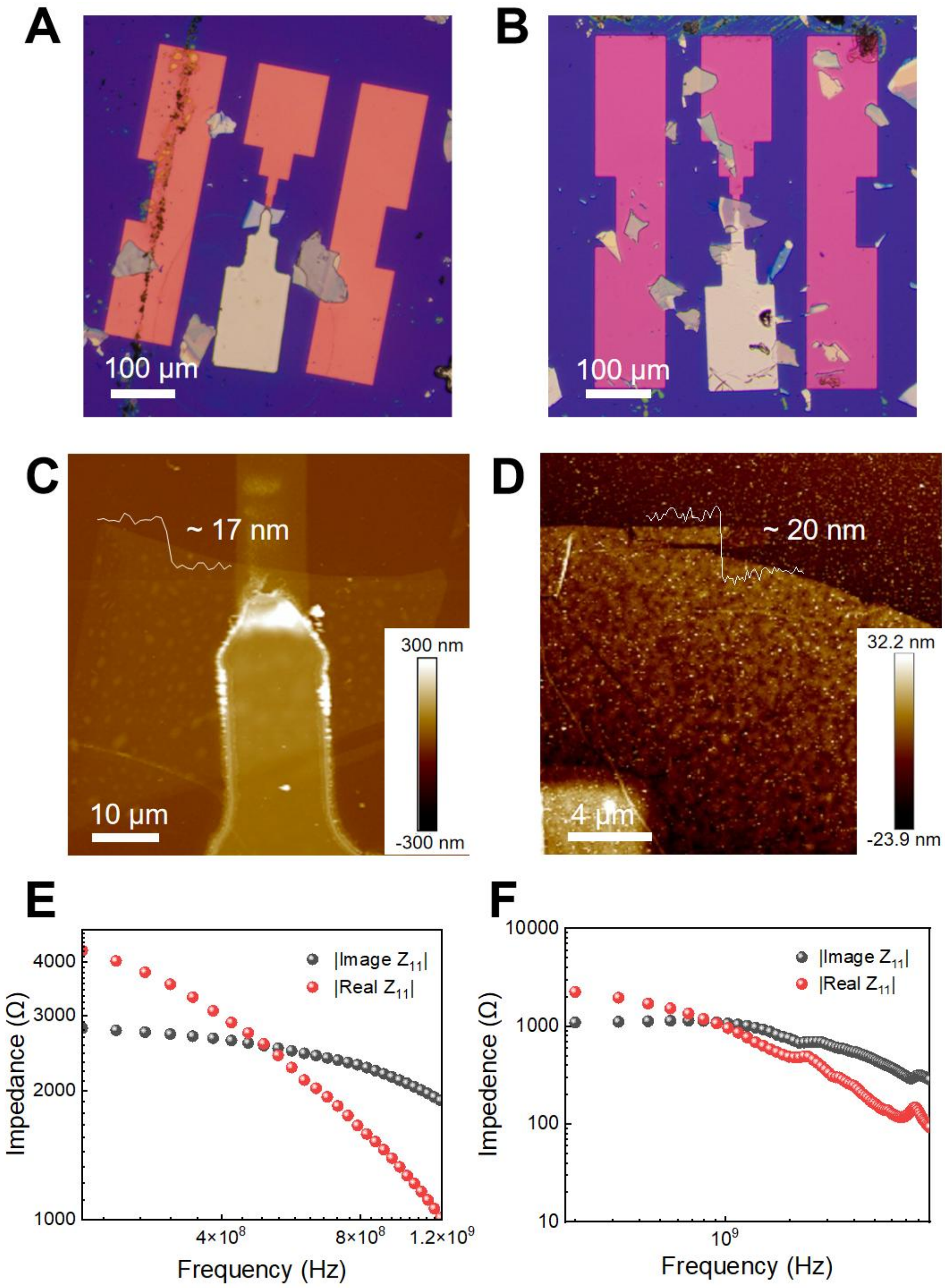

Fig. S7 Optical microscope images (A and B), AFM characterization (C and D), and electrical performance (E and F) of Device 1 and Device 2 summarized in Table S1.

The on-state S_{21} parameter reflects the insertion loss of the RF switch. As shown in the Fig S8, S_{21} remains relatively stable over the measured high-frequency range, increasing from approximately -6 dB at lower frequencies to around -5 dB at higher frequencies. Near the cut-off frequency of 57.5 GHz, the insertion loss is approximately -4.8 dB, indicating that the WSe_2 RF switch still maintains effective signal transmission at high frequencies. The relatively flat S_{21} response suggests stable on-state RF conduction, which can be attributed to the reduced series resistance enabled by the transferred Pd/WSe_2 vdW contact interface.Unlike memristive RF switches, our Schottky-diode-based RF switch is optimized for fast carrier transport and high-frequency response rather than low static power consumption. Therefore, this work is not intended to compete in terms of power consumption but to provide a complementary strategy for high-speed RF switching through low-damage transferred Pd/WSe_2 contacts.

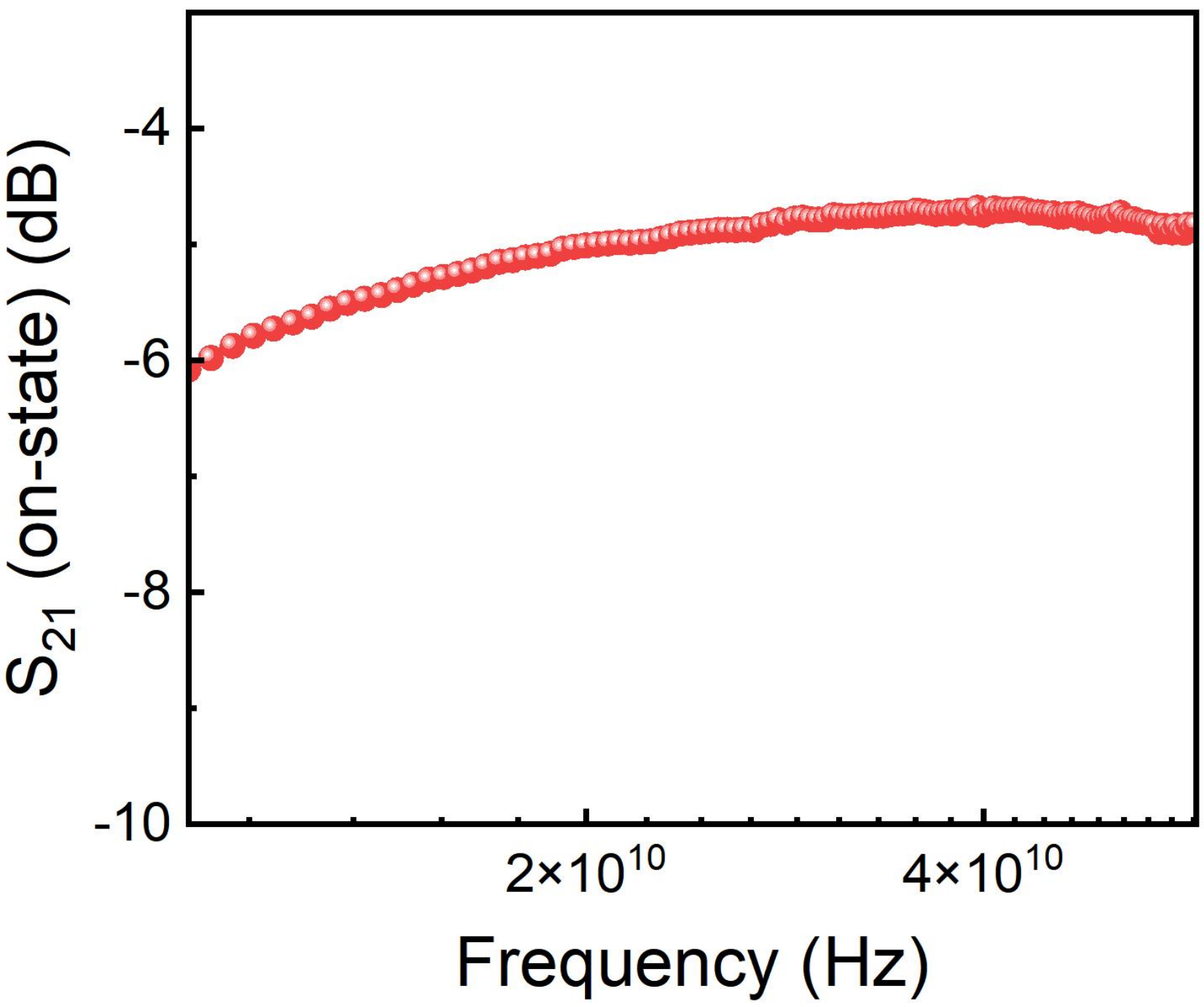

Fig. S8 Insertion loss (on-state) of the WSe_2 RF switch (56 nm-thick)

Table S2 Performance comparison of the reported graphene or TMD-based RF switches with this work

Material	Thickness (nm)	Cutoff Frequency (GHz)	On/Off Ratio	Junction Capacitance (pF)	Ref.
$MoSe_2$/graphene	60	37.3	6×10^5		[47]
MoS_2	1-3	4	10^3	0.038	[48]
MoS_2	1-3	10	10^2	0.004	[19]
WSe_2	50	27	10^8	0.1	[24]
WSe_2	12	10	-	-	[33]
$ReSe_2$	100	310	10^9-10^{10}	0.021	[35]
WSe_2	56	57.5	4.7×10^5	0.11	This work

High-fidelity transferred electrodes

Optical microscopy images (Fig. S9) of the electrode arrays supported on the PVA film demonstrate excellent pattern preservation after transfer. The electrode geometries remain highly uniform across the entire array, with well-defined edges and intact fine features. No noticeable deformation, fracture, or misalignment is observed, indicating that the PVA-assisted transfer process preserves the pre-patterned metal electrodes with high pattern fidelity. The uniform array integrity further confirms the mechanical stability and reproducibility of the transfer process.

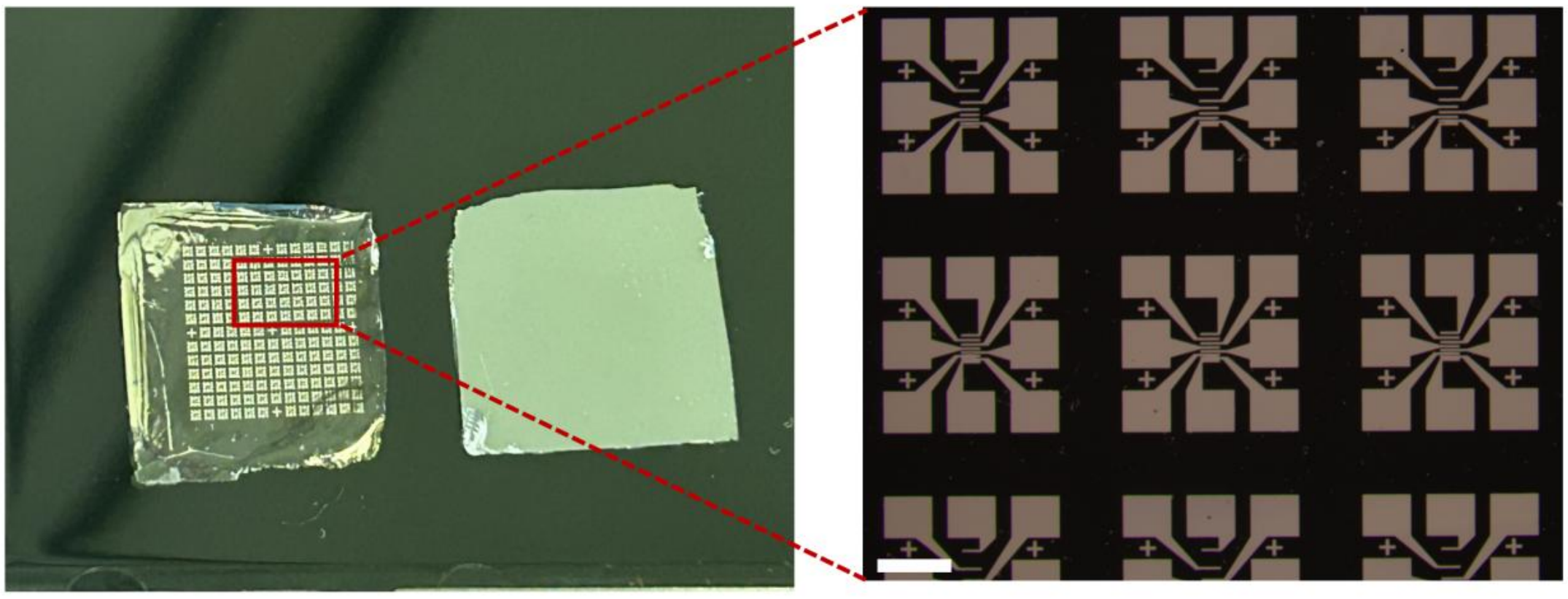

Fig. S9 Optical images of PVA-transferred electrode arrays (Scale bar, 200 μm).
